# Biliary Hyperkinesia: An Overlooked Cause of Right Upper Quadrant Pain

**DOI:** 10.7759/cureus.81875

**Published:** 2025-04-08

**Authors:** Amin Abu Hijleh, Filza Khalid, Karim Abdalbari, Shahzad Yousaf

**Affiliations:** 1 College of Medicine, Mohammed Bin Rashid University of Medicine and Health Sciences, Dubai, ARE; 2 General Surgery, Mediclinic Parkview Hospital, Dubai, ARE

**Keywords:** biliary dyskinesia, biliary hyperkinesia, case report, cholecystectomy, functional gallbladder disorder

## Abstract

Biliary colic, characterized by intermittent right upper quadrant (RUQ) abdominal pain, is a common clinical presentation worldwide. The most frequent underlying causes include acute or chronic cholecystitis and cholelithiasis. In cases where ultrasound findings are unremarkable, patients sometimes undergo a hepatobiliary iminodiacetic acid (HIDA) scan to evaluate gallbladder and biliary tree function. Traditionally, the results are categorized into two outcomes based on the gallbladder ejection fraction (GBEF): biliary dyskinesia or normal function. Biliary dyskinesia, or hypokinesia, is characterized by reduced gallbladder ejection fraction (GBEF), whereas biliary hyperkinesia involves abnormally elevated GBEF, reflecting excessive contractility and remaining poorly understood.

We present a case of a 55-year-old female with functional gallbladder disease manifesting as intermittent biliary colic. Initial investigations, including ultrasound and gastroscopy, were unremarkable. However, a HIDA scan revealed an elevated GBEF of 85% at 1 hour, consistent with biliary hyperkinesia. The patient experienced reproducible RUQ pain 40 minutes after consuming a fatty meal. She underwent laparoscopic cholecystectomy, which resulted in complete resolution of her symptoms.

A literature review highlights that many patients with normal imaging findings are diagnosed with biliary dyskinesia based on abnormal GBEF measurements from HIDA scans. While biliary hypokinesia is more commonly recognized, biliary hyperkinesia remains a rare entity. Surgical intervention, particularly laparoscopic cholecystectomy, has been shown to provide significant symptomatic relief in these patients.

In conclusion, many patients with biliary colic remain undiagnosed or are managed medically due to a lack of understanding of the underlying pathophysiology or insufficient diagnostic tools. As demonstrated in this case, we propose that patients with normal baseline investigations for biliary colic should undergo further evaluation, including HIDA scans, to rule out functional gallbladder disorders such as hypokinesia or hyperkinesia. Surgical treatment should be considered as a viable option for symptom relief, particularly in cases where pain causes significant distress and impairs quality of life.

## Introduction

Biliary colic, characterized by intermittent right upper quadrant (RUQ) abdominal pain, is a common clinical presentation worldwide. The most frequent underlying causes include acute or chronic cholecystitis and cholelithiasis [1]. However, in cases where ultrasound findings are unremarkable and no gallstones or structural abnormalities are detected, the diagnostic focus shifts to evaluating gallbladder function. This is typically achieved through a hepatobiliary iminodiacetic acid (HIDA) scan, which measures the gallbladder ejection fraction (GBEF) to assess biliary motility [2]. Traditionally, the results of a HIDA scan fall into two categories: normal gallbladder function or biliary dyskinesia. Functional gallbladder disorder, also referred to as biliary dyskinesia, represents a subset of gallbladder diseases marked by abnormal motility in the absence of gallstones or mechanical obstruction. This disorder encompasses two primary subtypes: biliary hypokinesia, defined by a reduced GBEF (<35%), and biliary hyperkinesia, characterized by an elevated GBEF (>65% to >80%, depending on the study) [3,4]. While biliary hypokinesia is well-documented and widely accepted, biliary hyperkinesia remains a poorly understood and underrecognized condition. Despite its rarity, biliary hyperkinesia can cause significant morbidity, with patients experiencing symptoms like those of biliary colic, including RUQ pain, nausea, and postprandial discomfort. 

Many patients with functional gallbladder disorders remain undiagnosed or are managed medically for extended periods due to unremarkable baseline imaging studies. For these individuals, persistent symptoms despite conservative treatment warrant further investigation with a HIDA scan to evaluate gallbladder function. Identifying biliary dyskinesia or hyperkinesia through this diagnostic tool can guide appropriate management, including consideration of cholecystectomy for definitive relief of symptoms. This approach is particularly relevant for patients with biliary hyperkinesia, as emerging evidence suggests that surgical intervention may provide significant symptomatic improvement in carefully selected cases [5]. Herein, we present a case of symptomatic biliary hyperkinesis, which was managed by undergoing laparoscopic cholecystectomy. The patient demonstrated complete symptom resolution following the cholecystectomy.

## Case presentation

**Figure 1:**
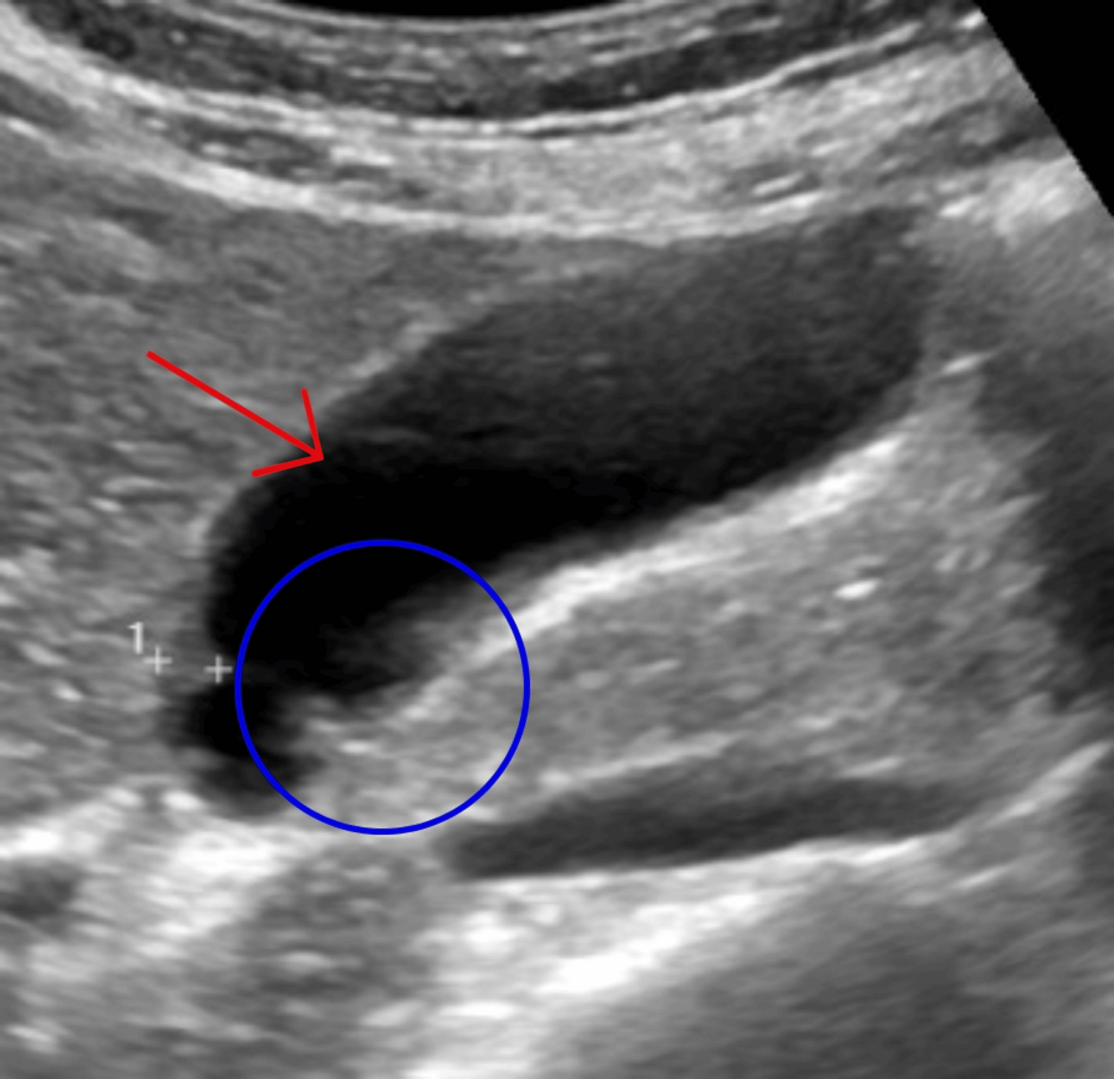
Ultrasonography of the gallbladder showing 2-3 polyps, no other abnormalities noted. Red arrow pointing to the gallbladder. Blue circle showing the gallbladder polyps.

We present a case of a 55-year-old South African female (BMI 24 kg/m²) who presented with recurrent episodes of severe, intermittent right upper quadrant (RUQ) abdominal pain over several years. The pain was described as agonizing, pulsating, and resembling a heartbeat, often accompanied by nausea, particularly after the consumption of fatty meals. These episodes initially occurred sporadically but gradually increased in frequency and severity over the past two years, becoming a daily occurrence. Despite multiple evaluations, including routine baseline investigations and ultrasonography (USG), no definitive diagnosis was established during the initial workup. The USG report showed a well-distended gallbladder without stones, sludge, or wall thickening, but with a few small (2-3 mm) polyps, with normal bile ducts and surrounding organs. Figure 1 shows the gallbladder from the ultrasound scan. The patient attempted dietary modifications, avoiding fatty foods, which provided temporary relief; however, symptoms consistently recurred upon reintroduction of such foods. 

The patient denied any associated jaundice, changes in bowel habits, or alterations in the color of her urine or stool, ruling out obstructive biliary pathology or hepatobiliary dysfunction. To further investigate her symptoms, an esophagogastroduodenoscopy (EGD) was performed to exclude gastritis or peptic ulcer disease. Histopathological examination of biopsy samples revealed no evidence of significant inflammation, intestinal metaplasia, dysplasia, or malignancy. Additional diagnostic workup, including a hepatobiliary iminodiacetic acid (HIDA) scan, demonstrated a markedly elevated gallbladder ejection fraction of 85% at the 1-hour mark, consistent with functional gallbladder disorder. Notably, the patient experienced reproducible RUQ pain approximately 40 minutes after consuming a fatty meal during the HIDA scan, further supporting the diagnosis. 

**Figure 2:**
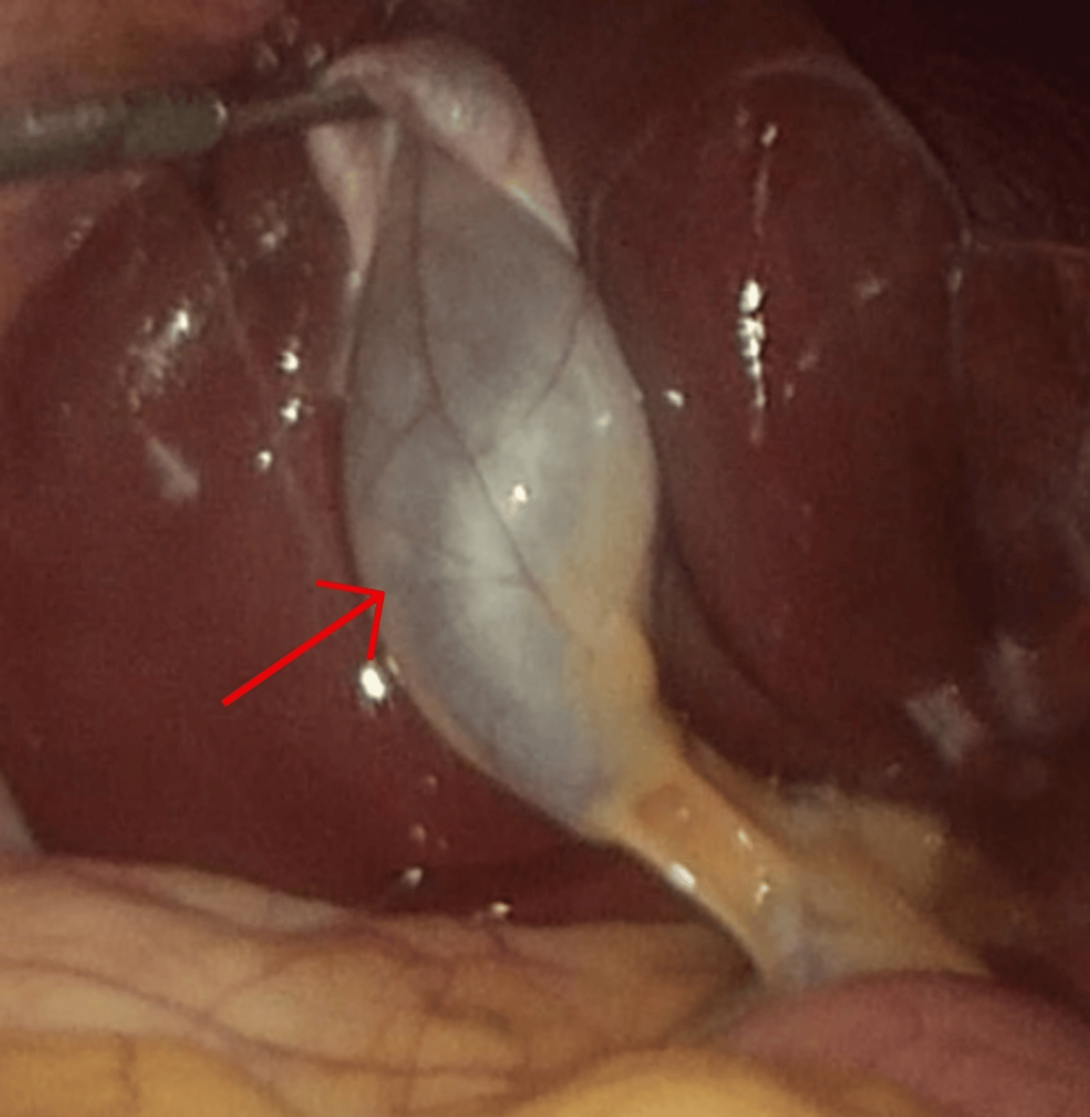
Intraoperative picture from laparoscopic cholecystectomy. Red arrow pointing to the gallbladder prior to resection.

**Figure 3:**
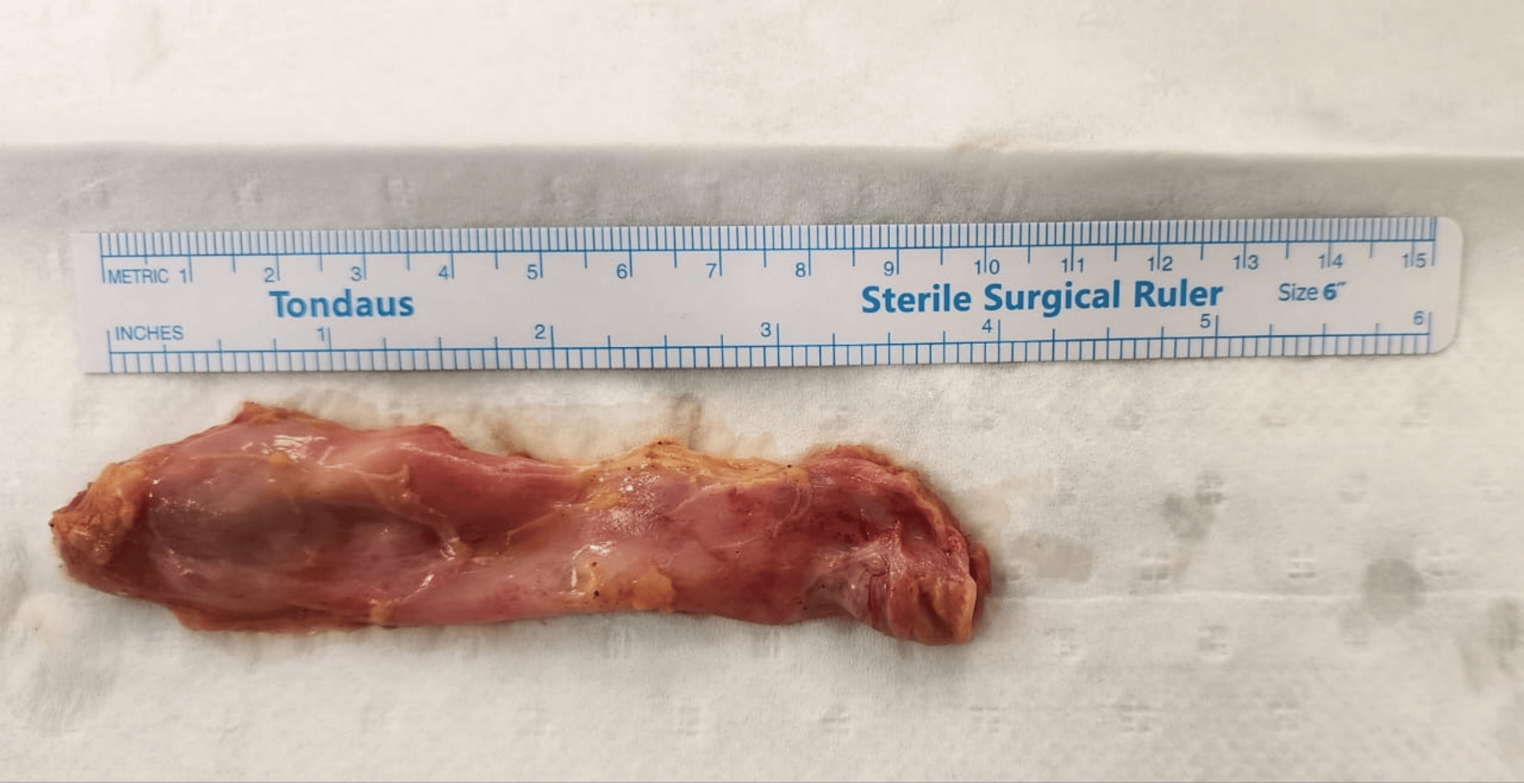
Acalculus gallbladder post-cholecystectomy

Given the persistent and debilitating nature of her symptoms, coupled with the absence of alternative explanations, the patient was scheduled for an elective laparoscopic cholecystectomy. The cystic duct and artery were carefully dissected, ligated, and divided, allowing for smooth gallbladder resection and removal. A relevant intraoperative photograph is displayed in Figure 2. The surgical procedure was performed without complications, and intraoperative findings revealed a grossly non-inflamed gallbladder as seen in Figure 3. The pathology report showed a thickened gallbladder wall due to lymphoplasmacytic infiltration in the lamina propria and fibrosis; Rokitansky Aschoff sinuses were noted. No features of dysplasia or malignancy were identified.

Following the laparoscopic cholecystectomy, the patient reported a resolution of her symptoms and was able to tolerate a regular diet symptom-free. Follow-up with the patient showed long-term improvement without symptom recurrence. This case highlights the diagnostic challenges associated with functional gallbladder disease and underscores the importance of a comprehensive evaluation, including functional imaging studies, in patients with atypical biliary symptoms. 

## Discussion

Biliary dyskinesia, particularly hypokinesia, has been extensively studied, but gallbladder hyperkinesia remains a rare and poorly understood condition. The prevalence of biliary hyperkinesia is thought to be lower than that of biliary hypokinesia, with limited epidemiological data available [6]. It is often diagnosed in patients presenting with biliary colic-like symptoms, such as right upper quadrant (RUQ) pain, nausea, and postprandial discomfort, in the absence of gallstones or structural abnormalities [4]. Although increasingly recognized in both adults and children, the lack of standardized diagnostic criteria and the overlap of symptoms with other functional gastrointestinal disorders make it challenging to determine the true prevalence of biliary hyperkinesia [7].

In our case, the patient was diagnosed with biliary hyperkinesia based on an elevated gallbladder ejection fraction (GBEF) of 85% at 1 hour during a hepatobiliary iminodiacetic acid (HIDA) scan. [3] The exact etiology of this condition remains unclear, but some studies suggest a potential role for cholecystokinin (CCK), a hormone produced by the small intestine that stimulates pancreatic enzyme secretion and gallbladder contraction [7]. It is hypothesized that increased CCK production or upregulation of CCK receptors may lead to excessive gallbladder contraction, causing intramural inflammation and a cholecystitis-like presentation [8]. Additionally, failure of the sphincter of Oddi to relax may exacerbate symptoms by creating a high-pressure biliary tract, further contributing to forceful contractions and pain [9].

The differential diagnosis for RUQ pain is broad and includes conditions such as cholecystitis, cholelithiasis, and cholangitis; less commonly, functional gallbladder disorders like biliary hyperkinesia may be the underlying cause [10]. Patients typically present with colicky RUQ pain that worsens after eating and may radiate to the back [11]. Initial evaluation often involves a RUQ ultrasound, which, if negative for gallstones, may prompt further investigation into functional gallbladder disease [7]. A HIDA scan is a valuable diagnostic tool, with a high GBEF supporting the diagnosis of biliary hyperkinesia [12]. The Rome IV criteria can also aid in diagnosing functional gallbladder disorders, particularly when extensive investigations fail to identify a mechanical cause for symptoms [13]. However, the lack of radiological evidence, combined with a low index of suspicion for functional gallbladder pathologies, could present a difficult challenge for physicians due to the similarity in clinical features between functional biliary disorders and other gallbladder pathologies; as a result, patients could suffer from this pain for years without a definite diagnosis. [14]

Most of the recent literature supports laparoscopic cholecystectomy as an effective treatment for biliary hyperkinesia. Cook et al. conducted a study at Ohio State University involving 14 patients, half of whom underwent laparoscopic cholecystectomy and half managed non-operatively. The study found that 100% of surgically treated patients experienced symptomatic relief, while none in the non-operative group improved, highlighting the efficacy of surgical intervention [15]. Similarly, Huckaby et al. at Saint Christopher’s Hospital for Children reported significant symptomatic relief in pediatric patients with biliary hyperkinesia following laparoscopic cholecystectomy, further supporting its use [16]. 

R.P. Maheswarappa et al. compared outcomes between a control group (n = 25) managed medically and a study group (n = 21) treated with cholecystectomy. Symptoms resolved in 86% of surgical patients, whereas 80% of medically managed patients experienced persistent pain, reinforcing the superiority of surgical intervention [17]. Gazzetta J. et al. reported similar findings in a cohort of 97 patients undergoing laparoscopic cholecystectomy for biliary hyperkinesia, with 90.9% of patients reporting symptom improvement or resolution [18]. Eltyeb et al. also demonstrated positive outcomes, with 303 out of 332 patients (91.3%) experiencing symptomatic improvement after surgery, although 29 patients (8.7%) did not benefit, suggesting that not all cases respond equally to cholecystectomy [19]. 

Despite these promising results, the use of laparoscopic cholecystectomy for biliary hyperkinesia remains controversial. Critics argue that hyperkinesia may represent a functional disorder rather than a structural pathology and removing the gallbladder may not address the underlying dysmotility or hypersensitivity [7]. Additionally, some patients experience persistent symptoms postoperatively, which may be attributed to overlapping functional gastrointestinal disorders such as irritable bowel syndrome (IBS) or functional dyspepsia, misdiagnosis, or postcholecystectomy syndrome.[12]. These concerns underscore the importance of careful patient selection and thorough preoperative counseling to ensure surgery is offered only to those likely to benefit [20]. 

On the contrary, while laparoscopic cholecystectomy is an effective treatment for many patients with biliary hyperkinesia, it is not universally successful. A study by Nasri et al. found that 61% of patients reported complete resolution of symptoms, and an additional 15% experienced partial relief, demonstrating a significant but not absolute rate of symptomatic improvement [8]. Therefore, a comprehensive diagnostic approach and individualized treatment plans are essential to optimize outcomes for patients with biliary hyperkinesia.

## Conclusions

In conclusion, current literature supports laparoscopic cholecystectomy as an effective treatment for biliary hyperkinesia, with high rates of symptomatic relief. However, the condition's functional nature and a minority of patients showing no improvement of symptoms post-surgery highlight the need for further research to refine diagnostic criteria, understand the pathophysiology, and optimize patient selection.
